# Social anxiety disorder in genuine halitosis patients

**DOI:** 10.1186/1477-7525-9-94

**Published:** 2011-11-03

**Authors:** Takashi Zaitsu, Masayuki Ueno, Kayoko Shinada, Fredrick A Wright, Yoko Kawaguchi

**Affiliations:** 1 Department of Oral Health Promotion, Graduate School of Medical and Dental Sciences, Tokyo Medical and Dental University, Japan; 2 Department of Oral Health Care Promotion, School of Oral Health Care Sciences, Faculty of Dentistry, Tokyo Medical and Dental University, Japan; 3 Centre for Oral Health Strategy, New South Wales, Australia

## Abstract

**Background:**

There is a possibility that genuine halitosis patients' anxiety do not recover after oral malodor treatment due to their social anxiety disorder. The objective of this study was to investigate the influence of social anxiety disorder on the level of anxiety in genuine halitosis patients before and after treatment for oral malodor.

**Methods:**

The subjects were 262 genuine halitosis patients who visited the Fresh Breath Clinic from March, 2008 to October, 2009. The subjects who had score 2 or higher by the organoleptic test were diagnosed as genuine halitosis patients. Gas chromatography (GC) was conducted before and after oral malodor treatment for the oral malodor measurement. Based on their risk of social anxiety disorder, subjects were divided into low- and high-risk groups using the Liebowitz Social Anxiety Scale (LSAS). The questions related to oral malodor and the clinical oral examination were both conducted before oral malodor treatment. The level of anxiety before and after oral malodor treatment was evaluated using the Visual Analogue Scale of Anxiety (VAAS).

**Results:**

More than 20% of subjects had a score of 60 or more on the LSAS (high LSAS group). The mean age and the percentage of females were significantly higher in the high LSAS group compared to the low LSAS group. The high LSAS group was more likely to have problems associated with oral malodor and to adopt measures against oral malodor compared to the low LSAS group. The mean concentrations of H_2_S and CH_3_SH by GC significantly decreased after the oral malodor treatment in both LSAS groups. VAAS scores also significantly decreased after treatment in both LSAS groups. The logistic regression analysis indicated that the high LSAS group had a 2.28 times higher risk of having a post-VAAS score of 50 or more compared to the low LSAS group.

**Conclusions:**

This study revealed that genuine halitosis patients with a strong trait of social anxiety disorder have difficulty overcoming their anxiety about oral malodor. Oral malodor treatment of genuine halitosis patients requires not only regular oral malodor treatment but also attention to social anxiety disorder.

## Background

Several studies reveal that social anxiety disorder is the most common anxiety disorder. It usually has an early onset, and has serious effects on social interactions and quality of life [1,2]. The Diagnostic and Statistical Manual, Fourth Edition (DSM-IV) of the American Psychiatric Association defines social anxiety disorder as a persistent fear in one or more social or performance situations whenever the person is exposed to unfamiliar people or to possible scrutiny by others [3]. In the United States, social anxiety disorder has the third highest prevalence among psychological diseases after depression and alcohol dependence [4]. Excessive anxiety in social situations causes considerable distress and impairs carrying out daily activities [5].

Previous research revealed that pseudohalitosis patients have higher scores on some items of the Liebowitz Social Anxiety Scale (LSAS), a questionnaire to evaluate the social anxiety disorder, than genuine halitosis patients [6]. Moreover, generalized social anxiety disorder was observed in 19.5% of genuine halitosis patients, and 27.9% of the pseudohalitosis patients, for a combined 21.8% of all halitosis patients. These findings suggest that there is a relationship between the type of patient with oral malodor and social anxiety disorder.

According to the classification of halitosis by Yaegaki et al., genuine halitosis is defined as "obvious malodor with intensity beyond a socially acceptable level is perceived", and pseudohalitosis is defined as "obvious malodor is not perceived by others, although the patient stubbornly complains of its existence. [7,8]" Some research indicates that pseudohalitosis patients have a stronger tendency for depression compared with genuine halitosis patients [9,10]. It has also been suggested that pseudohalitosis is related to both their somatic and emotional status, and that psychological disorders are strongly associated with the halitosis classification. Thus, pseudohalitosis patients receive instruction, education and counseling as part of their treatment [11].

However, the counseling procedure for genuine halitosis patients has not been examined thoroughly. It is often the case that the treatment of the genuine halitosis patient ends when regular oral malodor treatment ends. Because their mental aspect is not fully addressed, some genuine halitosis patients do not improve their anxiety level and QOL despite amelioration of their oral malodor. Some research suggests that, like pseudohalitosis patients, genuine halitosis patients may also have associated psychological problems, such as social anxiety disorder [7,12], that may be overlooked. The hypothesis in this study is that some genuine halitosis patients may have social anxiety disorder that hinders their recovery from oral malodor related anxiety. If a relationship between social anxiety disorder and the worry or uneasiness of oral malodor patients is established, the necessity for oral malodor treatment to take social anxiety disorder into consideration will be clarified.

Therefore, the purposes of this study were to examine the influence of social anxiety disorder on oral malodor-related anxiety before and after the treatment of the oral malodor.

## Methods

### Study subjects

Patients who visited or were referred to the Fresh Breath Clinic at the Dental Hospital of Tokyo Medical and Dental University between March, 2008 and October, 2009 were invited to join the study. After being told the nature of the research, two hundred and sixty-two patients (83 males and 179 females, mean age: 51.9 ± 14.3 years, age range 16-83 years), diagnosed with genuine halitosis, signed the informed consent form and participated in the study. The study protocol was approved by the Tokyo Medical and Dental University Ethics Committee.

### Study Instruments

A self-administered questionnaire was administered to all subjects at the initial visit. The questionnaire consisted of 1) Questions related to oral malodor, 2) The Liebowitz Social Anxiety Scale (LSAS) [13] and 3) The Visual Analogue Scale of Anxiety (VAAS) [14,15]. Following the questionnaire, oral malodor and oral health status were assessed. Oral malodor was measured by specialist dentists at the clinic. The oral malodor treatment was based on the Treatment Need (TN) by classification of Halitosis by Yaegaki et al. [7,11,16]. After the completion of treatment for oral malodor, we conducted the VAAS again.

#### 1. Questionnaire

(1) Questions related to oral malodor

The following five questions consisted of: 1) "How did you first notice your bad breath?", 2) "What do you see as the problem with your having bad breath?", 3) "Is there anything you do to decrease your bad breath?", 4) " Are you anxious about others' smells?", 5) "Are you sensitive to the smell?", and 6)" Are you anxious about body odor?"

(2) Liebowitz Social Anxiety Scale (LSAS)

The LSAS contains 24 situational questions (13 performance and 11 social interaction items), to which subjects respond on a 4-point scale for both fear/anxiety and avoidance sections [13,17]. The scale range of fear/anxiety was from 0 to 3 (0 = none, 1 = mild, 2 = moderate, and 3 = severe) and avoidance was from 0 to 3 (0 = never, 1 = occasionally, 2 = often, and 3 = usually). Total fear/anxiety and total avoidance scores are both 0-72, and thus, the total LSAS score falls between 0-144.

The Japanese version of the scale (LSAS-J) was used in this study [18]. The cutoff value of the LSAS to diagnose generalized social anxiety disorder is 60 [19,20]. Therefore, we placed subjects with a total LSAS score of 60 or higher in the high LSAS group and subjects with a total LSAS score of 59 or lower in the low LSAS group.

(3) Visual Analogue Scale of Anxiety (VAAS)

The VAAS was conducted to evaluate patients' anxiety before treatment (pre-VAAS) and after treatment (post-VAAS). The VAAS is a 10 cm-long horizontal line, in which subjects mark their anxiety level with a ballpoint pen. The minimum score is 0 which means "no anxiety at all" and the maximum is 100 representing "worst anxiety imaginable [21]." We placed subjects with VAAS scores of less than 50 in the "low VAAS group", and those with scores of 50 or more in the "high VAAS group".

#### 2. Oral malodor assessment

We conducted two methods, the Organoleptic test (OT) and Gas chromatography (GC) for the oral malodor measurement. Measurements were conducted between 9 and 11 o'clock in the morning because morning breath odor has been used as a standard mouth breath for oral malodor [22]. We advised patients not to have food or drink, and to refrain from their usual oral hygiene practice on the morning of the oral malodor assessment. To exclude confounding smells, we instructed patients to stop eating strong-smelling foods for at least 48 hours before the oral malodor assessment, stop using strong-scented perfumes for 24 hours, and stop smoking or drinking alcohol for 12 hours prior to the assessment.

(1) Organoleptic Test (OT)

We used the OT as the clinical measurement of oral malodor. The OT was conducted after subjects had closed their mouth for 3 minutes while breathing through their nose. The OT was performed by 7 dentists, who were calibrated with the T&T Olfactometer (Daiichi Yakuhin Sangyo Co. Tokyo, Japan), an odor solution kit for examining the olfactory sense [23,24]. Two judges rated the malodor on a 0-5 scale where a score of 0 = absence of odor, 1 = barely appreciable odor, 2 = slight malodor, 3 = moderate malodor, 4 = strong malodor, and 5 = severe malodor [25-27]. If the judges gave different scores, the mean score represented the score for the subject. Subjects who were scored 2 or higher by the OT were diagnosed as having genuine halitosis.

(2) Gas chromatography (GC)

A GC-8A gas chromatograph (Shimadzu, Kyoto, Japan) equipped with a flame photometric detector was used for the GC analysis. It has an auto-injection system with a 10 ml Teflon (Du Pont, Tokyo, Japan) sample loop and a column packed with 25% 1, 2, 3-tris (2-cyanoethoxy) propane on an 80/100 mesh Shimalite AW-DMCS-ST support system at 60°C. The Teflon tube was directly inserted into the oral cavity of a patient through the lips and teeth for the malodor measurement, and 20 mL of mouth air was aspirated with a syringe connected to the outlet of the auto-injector. Following the aspiration, a 10 mL sample of air was transferred to the column and chromatographed by a sulfur chemiluminescence detector that specifically responded to sulfur. The volatile sulfur compounds (VSCs) gases, H_2_S and CH_3_SH, were determined by their characteristic retention times, and quantities were calculated by comparing their peak areas with those of dilutions of standard gases of H_2_S and CH_3_SH that were prepared with a PD-1B permeater (Gastec Company, Kanagawa, Japan). Outcomes were shown as concentrations of H_2_S and CH_3_SH (ng/10 mL). Based on the olfactory threshold levels (H_2_S > 1.5 ng/10 mL and CH_3_SH > 0.5 ng/10 mL) proposed by Tonzetich [28], patients were classified as either normal or having malodor.

#### 3. Oral health status

The clinical oral examination included an assessment of the number of teeth present, number of decayed teeth, periodontal pocket depth (PPD), bleeding on probing (BOP), oral hygiene and volume of resting saliva.

Standardized clinical criteria were based on the W.H.O. format [29]. We examined the PPD using a dental mirror and a periodontal probe. The deepest PPD was recorded by probing circumferentially around the tooth. The average value of PPD of all teeth was used as the representative value for the person. We checked presence of BOP on each tooth while measuring the PPDs. Oral hygiene was evaluated using the Silness-Löe plaque index (PI) on six index teeth [30]. The score ranged from 0 to 3 where a score of 0 = no plaque, a score of 1 = a film of plaque adhering to the free gingival margin and adjacent area of the tooth with the plaque visible when using the probe on the tooth surface, a score of 2 = moderate accumulation of soft deposits within the gingival pocket, or the tooth and gingival margin that could be seen with the naked eye, and a score of 3 = abundance of soft matter within the gingival pocket or on the tooth and gingival margin. The average value of all tooth surfaces was recorded as the plaque index score.

We collected resting saliva by letting subjects spit pooled saliva into a cup for 5 minutes. The resting salivary flow rate was calculated as amount per minute (mL/min).

### Statistical analysis

Student t-tests and Chi-squared tests were used to compare the means or distributional differences of age, gender, oral health status, questions related to oral malodor and VAAS between the low LSAS and high LSAS groups. Paired t-tests were conducted to detect differences of mean concentrations of VSC gases and VAAS scores before and after treatment. Values of oral health status were recorded for multivariate statistical analysis. We categorized patients as having either 19 or fewer teeth present or 20 or more teeth present, and separately categorized as having either 0 decayed teeth or 1 or more decayed teeth. For PPD, BOP, and PI, patients were categorized into low and high groups by the median. For salivary flow rate, patients were categorized as having less than 0.1 mL/min and 0.1 mL/min or more. The concentrations of H_2_S and CH_3_SH were dichotomized using the threshold levels.

ANCOVA was performed with the post-VAAS score as the dependent variable, and the two LSAS groups as independent variables after adjusting for age, gender, pre-VAAS score and the concentration of VSC gases after the treatment. We conducted logistic regression analysis with the post-VAAS score as the dependent variable, and age, gender, LSAS, oral health status, pre-VAAS score, and the concentration of VSC gases after treatment as independent variables.

All tests were conducted at the 5% significance level. SPSS statistical software package was used for all analyses (SPSS 18.0J; SPSS Japan, Tokyo, Japan).

## Results

### Characteristics of the two LSAS groups

The mean LSAS score was 38.9 ± 28.3. The proportion of subjects in the high LSAS group was 22.9% (N = 60), and in the low LSAS group was 77.1% (N = 202). The mean age and the percentage of females were significantly higher in the high LSAS group (55.5 ± 14.5, 85.0%, respectively) compared to the low LSAS group (50.8 ± 14.0, 63.4%, respectively) (P = 0.031, P = 0.001, respectively).

### Oral malodor

The mean concentrations of H_2_S before the oral malodor treatment were 6.44 ± 5.29 in the low LSAS group and 6.98 ± 5.39 in the high LSAS group, and after treatment the concentrations were 0.54 ± 0.81 in the low LSAS group and 0.42 ± 0.79 in the high LSAS group. Both groups had significantly decreased concentrations of H_2_S after treatment (P < 0.001). The mean concentrations of CH_3_SH before the oral malodor treatment were 2.31 ± 2.21 in the low LSAS group and 2.37 ± 2.18 in high LSAS group, and after the treatment the concentrations were 0.17 ± 0.26 in the low LSAS group and 0.11 ± 0.23 in the the high LSAS group. Both groups had significantly decreased concentrations of CH_3_SH after treatment (P < 0.001).

### Oral health status

As shown in Table 1 the high LSAS group had a significantly lower number of teeth present and a significantly lower plaque index score compared with the low LSAS group. There were no significant differences in the number of decayed teeth, PPD, BOP or volume of resting saliva between the two groups.

**Table 1:** Clinical characteristics of subjects by LSAS score

	*LSAS*				
	*Low (-59)*		*High (60-)*		
	*(N = 202)*		*(N = 60)*		*P value*
	*Mean*	*SD*	*Mean*	*SD*	
Number of teeth present	26.1	4.0	24.2	5.4	0.014*
Number of decayed teeth	0.4	1.5	0.1	0.5	0.053
PPD (mm)	2.4	0.5	2.5	0.4	0.604
BOP(teeth)	4.3	5.2	4.1	4.1	0.765
Plaque Index	0.5	0.4	0.4	0.3	0.042*
Salivary flow rate (mL/min)	0.3	0.3	0.3	0.3	0.295

* Significant at p < 0.05

### Questions related to oral malodor

There was no significant distributional difference of responses to the question "How did you first notice your bad breath?" between the two LSAS groups (Table 2). For the question "What do you see as the problem with your having bad breath?", the percentages of subjects who answered "Cannot talk with people", "Cannot act with people", "Cannot be active and become negative about everything", "Cannot concentrate" or "Cannot make close friends" were significantly higher in the high LSAS group compared with the low LSAS group. The percentages of subjects who answered "Brush teeth many times" and "Cover my mouth while talking with people" to the question "Is there anything you do to decrease your bad breath?" were significantly higher in the high LSAS group than in the low LSAS group. The proportion of subjects who answered "yes" to the question "Are you anxious about body odor?" was significantly higher in the high LSAS group than in the low LSAS group.

**Table 2:** Oral malodor related worries and problems by LSAS score

	*LSAS*		
	*Low (-59)*	*High (60-)*	*P value*
	*(N = 202)*	*(N = 60)*	
1. How did you first notice your bad breath?(multiple answers)			
You noticed it by yourself	24.8%	21.7%	0.732
You have ever been told by others you have bad breath	42.6%	56.7%	0.057
You suspect that you have bad breath based upon the actions of other persons	67.3%	65.0%	0.716
2. What do you see as the problem in your having bad breath? (multiple answers)			
Nothing in particular	14.4%	6.7%	0.127
Cannot talk with people	48.0%	68.3%	0.008*
Cannot act with people	14.4%	46.7%	< 0.001*
People avoid me	6.9%	10.0%	0.415
Cannot be active and become negative about everything	37.6%	71.7%	< 0.001*
Cannot concentrate	10.4%	23.3%	0.016*
Cannot make close friends	1.5%	11.7%	0.002*
3. Is there anything you do to decrease your bad breath? (multiple answers)			
Nothing in particular	5.0%	3.3%	0.739
Brush my teeth many times	66.3%	81.7%	0.025*
Use mouth-rinsing solution/chewing gum/mouth drops	69.8%	68.3%	0.874
Decrease the frequency of meals or snacks	1.0%	5.0%	0.081
Cover my mouth while talking with people	31.7%	48.3%	0.021*
Try to find out the cause of bad breath by visiting many hospitals and undergoing examinations	13.9%	21.7%	0.158
Clean my tongue	63.9%	65.0%	0.619
4. Are you anxious about others' smells?	75.7%	66.7%	0.183
5. Are you sensitive to the smell?	59.4%	60.0%	> 0.999
6. Are you anxious about the body odor?	59.4%	80.0%	0.003*

* Significant at p < 0.05

### VAAS

Pre-VAAS scores were significantly higher in the high LSAS group (72.0 ± 22.5) compared to the low LSAS group (60.2 ± 28.5; P = 0.001). Post-VAAS scores were also significantly higher in the high LSAS group (52.0 ± 25.8) compared to the low LSAS group (25.0 ± 20.8; P = 0.005). Moreover, VAAS scores significantly decreased after treatment in both groups (P < 0.001). The proportion of subjects with a pre-VAAS score of 50 or higher was significantly higher in the high LSAS group (88.0%) compared to the low LSAS group (70.8%; P = 0.006), and after treatment the proportion was still significantly higher in the high LSAS group (30.0%) compared to the low LSAS group (15.8%; P = 0.023).

ANCOVA showed that the high LSAS group had a higher post-VAAS score (30.8 ± 2.7SE) compared to the low LSAS group (23.2 ± 1.4SE, P = 0.016). The logistic regression analysis showed that the high LSAS group had a 2.28 times higher risk of having a post-VAAS score of 50 or more compared to the low LSAS group (P = 0.037). Moreover, the high pre-VAAS group had a 7.09 times higher risk of having a post-VAAS score of 50 or more compared to the low pre-VAAS group (P = 0.002) (Table 3).

**Table 3:** Logistic regression analysis of post-VAAS

	*Group*	*N*	*Odds ratio*	*P value*
Age	-39	55	reference	
	40-59	120	0.72	0.475
	60-	87	0.47	0.158
Gender	Male	83	Reference	
	Female	179	0.68	0.326
LSAS	-59	202	Reference	
	60-	60	2.28	0.037*
Teeth present (teeth)	20-	24	Reference	
	-19	220	0.85	0.787
Decayed teeth (teeth)	0	227	reference	
	1-	35	0.23	0.063
PPD (mm)	Low	132	reference	
	High	130	0.55	0.128
BOP (teeth)	Low	160	reference	
	High	102	1.53	0.298
Plaque Index	Low	130	reference	
	High	132	1.11	0.797
Salivary flow rate (mL/min)	< 0.1	42	reference	
	> = 0.1	220	1.16	0.779
Pre-VAAS	< 50	66	reference	
	> = 50	196	7.09	0.002*
H_2_S (ng/10mL) (after treatment)	< 1.5	238	reference	
	> = 1.5	23	4.14	0.086
Ch_3_SH (ng/10mL) (after treatment)	< 0.5	237	reference	
	> = 0.5	24	0.31	0.199

* Significant at p < 0.05

## Discussion

This research indicates that those genuine halitosis patients with a strong trait of social anxiety disorder have difficulty overcoming their anxiety about oral malodor. The relationship between social anxiety disorder and some other diseases or disorders, such as alcohol dependency or strabismus, has been investigated [17,31]. Our previous study showed a close relationship between social anxiety disorder and the classification of halitosis [6]. However, the current study is the first to investigate the relationship between the level of social anxiety disorder and the amount of improvement of anxiety contingent on oral malodor.

The LSAS is recognized by the International Consensus Group on Depression and Anxiety as the gold standard for evaluating the clinical impact of social anxiety disorder in an individual [32]. The LSAS has been translated into many languages besides English [33,34], and its reliability and validity have been confirmed. The Japanese version of the LSAS also shows high reliability and verified validity [18]. The VAAS has been used in several studies to measure the degree of anxiety in both anxiety-disorder and healthy subjects. It has been proven to be a valid method for the measurement of anxiety and is highly sensitive to changes [14,15].

This study revealed that 22.9% of genuine halitosis patients had a tendency for general social anxiety disorder. This percentage is almost the same as the 19.5% reported in our previous study [6]. The high LSAS group was predominantly female. According to Turk et al., females tend to have a higher risk for social anxiety disorder [35]. Social anxiety disorder is reported to be more likely to develop in younger people [36,37]. However, in this study the mean age of subjects in the high LSAS group was higher than that in the low LSAS. There is the fact that female subjects who were more dominant in the high LSAS group were older, and it might have influenced the results.

Regarding oral health status, there was a significant difference in the number of teeth present; the high LSAS group had less teeth present compared to the low LSAS group. This also reflects the age difference between the two LSAS groups, the high LSAS group being older. The excellent plaque control in the high LSAS group may be attributed to their high motivation for brushing to prevent oral malodor.

The high LSAS group was more likely to have problems associated with oral malodor and to adopt countermeasures against oral malodor compared to the low LSAS group. Moreover, the high LSAS group felt anxious not only about oral malodor but also about body malodor.

Most halitosis patients greatly improved after receiving the oral malodor treatment that includes plaque control instruction, tongue cleaning, and mouth rinses. The concentrations of VSCs were also significantly reduced in both LSAS groups. However, in the short-term, anxiety remained significantly higher in the high LSAS group even after the treatment compared with the low LSAS group. About 30% of the high LSAS group still had a VAAS score of 50 or higher after treatment, a percentage almost twice as high as that of the low LSAS group. Moreover, after controlling for age, gender, oral health status, pre-VAAS, and the concentration of VSC gases, the presence of social anxiety disorder greatly influenced the anxiety after the oral malodor treatment. Thus, social anxiety disorder should be considered in regular oral malodor treatment. There is the study of Rosenberg et al. which revealed that the people with complaint of oral malodor were not capable of sensing reductions in oral malodor 1 year following original assessment, even though, from a clinical standpoint, improvements have taken place [38]. This result was similar to our study that anxiety did not improve in the high LSAS group after the treatment.

It is of great significance that improvement of anxiety about oral malodor will be insufficient in genuine halitosis patients if they have a social anxiety disorder. This finding suggests that anxiety about oral malodor in genuine halitosis patients will only be improved by the treatment of the social anxiety disorder in addition to the oral malodor treatment. Some treatment regimens for social anxiety disorder, such as cognitive-behavioral therapy [39,40] or medical treatment [41-44], have been introduced. Dentists in oral malodor clinics must cooperate with staff from other departments and test for social anxiety disorder in addition to performing regular oral malodor treatment. And for further research and treatment, we have to consider the anxiety traits in Japanese social anxiety disorder patients. The previous study revealed that Japanese psychiatric patients diagnosed with social anxiety disorder tend to have "relationship fears" unique to the Japanese [45]. This result corresponded to the result in this study that the high LSAS group worried about communications. It is important for the treatment and research which take cultures and societies in Japan into consideration.

The limitation of this research is that the VAAS was not conducted in the long term. It was conducted only before and after the treatment. We should follow the VAAS in long term treatment. And this study targeted only genuine halitosis patients, despite almost 30% of the patients who visit oral malodor clinics are pseudohalitosis patients [6]. Therefore, it remains necessary to evaluate how social anxiety disorder and anxiety of pseudohalitosis patients changes after oral malodor treatment. Moreover, it will be essential to assess whether any change in anxiety for oral malodor occurs when the social anxiety disorder is treated.

## Conclusions

Our study revealed that those genuine halitosis patients with a strong trait of social anxiety disorder have difficulty overcoming their anxiety about oral malodor. Oral malodor treatment of genuine halitosis patients requires not only regular oral malodor treatment but also attention to social anxiety disorder. The implication of this research is that this is the first study to investigate the relationship between social anxiety disorder and improvement of anxiety by oral malodor treatment. And this result will contribute to construct new system for oral malodor treatment.

## Competing interests

The authors declare that they have no competing interests.

## Authors' contributions

TZ has made substantial contribution to the study conception and design. TZ, MU, KS and YK implemented this study and participated in the acquisition, analysis and interpretation of data. TZ, MU, KS, FACW and YK have been intimately involved in drafting and editing the manuscript. All authors read and approved the final manuscript.

## References

[B1] Kessler RC The impairments caused by social phobia in the general population: implications for intervention Acta psychiatrica Scandinavica Supplementum 2003 417 19 27 10.1034/j.1600-0447.108.s417.2.x 12950433

[B2] Magee WJ Eaton WW Wittchen HU McGonagle KA Kessler RC Agoraphobia, simple phobia, and social phobia in the National Comorbidity Survey Archives of General Psychiatry 1996 53 2 159 168 8629891 10.1001/archpsyc.1996.01830020077009

[B3] Diagnostic and statistical manual of mental disorders American Psychiatric Association 2000 4 Washington, DC Text revision

[B4] Kessler RC McGonagle KA Zhao S Nelson CB Hughes M Eshleman S Lifetime and 12-month prevalence of DSM-III-R psychiatric disorders in the United States. Results from the National Comorbidity Survey Archives of General Psychiatry 1994 51 1 8 19 8279933 10.1001/archpsyc.1994.03950010008002

[B5] Wittchen HU The many faces of social anxiety disorder International Clinical Psychopharmacology 2000 15 Suppl 1 7 12 10.1097/00004850-200007001-00003 10994677

[B6] Zaitsu T Ueno M Shinada K Wright FAC Kawaguchi Y Relationship between Social Anxiety Disorder and Halitosis International Journal of Clinical Dentistry 2011 6 2 63 71

[B7] Yaegaki K Coil JM Examination, classification, and treatment of halitosis; clinical perspectives Journal of the Canadian Dental Association 2000 66 5 257 261 10833869

[B8] Yaegaki K Coil JM Genuine halitosis, pseudo-halitosis, and halitophobia: classification, diagnosis, and treatment Compendium of Continuing Education in Dentistry 2000 21 10A 880 886 8-9; quiz 90 11908365

[B9] Oho T Yoshida Y Shimazaki Y Yamashita Y Koga T Psychological condition of patients complaining of halitosis Journal of Dentistry 2001 29 1 31 33 10.1016/S0300-5712(00)00057-9 11137636

[B10] Suzuki N Yoneda M Naito T Iwamoto T Hirofuji T Relationship between halitosis and psychologic status Oral Surgery, Oral Medicine, Oral Pathology, Oral Radiology, and Endodontology 2008 106 4 542 547 10.1016/j.tripleo.2008.03.009 18602310

[B11] Miyazaki H Arao M Okamura K Kawaguchi Y Toyofuku A Hoshi K Tentative classification of halitosis and its treatment needs.(Japanese) Niigata Dental Journal 1999 32 7 11

[B12] Yaegaki K Rosenberg M editor Bad Breath Research Perspectives Tel-Aviv: Ramot Publishing-Tel Aviv University 1995 87 108

[B13] Liebowitz MR Social phobia Modern Problems of Pharmacopsychiatry 1987 22 141 173 2885745 10.1159/000414022

[B14] Gift AG Visual analogue scales: measurement of subjective phenomena Nursing research 1989 38 5 286 288 2678015

[B15] Hornblow AR Kidson MA The visual analogue scale for anxiety: a validation study The Australian and New Zealand journal of psychiatry 1976 10 4 339 341 10.3109/00048677609159523 1071419

[B16] Coil JM Yaegaki K Matsuo T Miyazaki H Treatment needs (TN) and practical remedies for halitosis International dental journal 2002 52 Suppl 3 187 191 12090450 10.1002/j.1875-595x.2002.tb00922.x

[B17] Evren C Sar V Dalbudak E Oncu F Cakmak D Social anxiety and dissociation among male patients with alcohol dependency Psychiatry research 2009 28,165 3 273 280 10.1016/j.psychres.2007.10.016 19162331

[B18] Asakura S Inoue S Sasaki F Sasaki Y Kitagawa N Inoue T Reliability and validity of the Japanese version of the Liebowitz Social Anxiety Scale Seishin Igaku (Clinical Psychiatry) 2002 10,44 10 1077 1084

[B19] Mennin DS Fresco DM Heimberg RG Schneier FR Davies SO Liebowitz MR Screening for social anxiety disorder in the clinical setting: using the Liebowitz Social Anxiety Scale Journal of anxiety disorders 2002 16 6 661 673 10.1016/S0887-6185(02)00134-2 12405524

[B20] Rytwinski NK Fresco DM Heimberg RG Coles ME Liebowitz MR Cissell S Screening for social anxiety disorder with the self-report version of the Liebowitz Social Anxiety Scale Depression and anxiety 2009 26 1 34 38 10.1002/da.20503 18781659

[B21] van Duinen M Rickelt J Griez E Validation of the electronic Visual Analogue Scale of Anxiety Progress in neuro-psychopharmacology and biological psychiatry 2008 15,32 4 1045 1047 10.1016/j.pnpbp.2008.02.002 18343008

[B22] van Steenberghe D Avontroodt P Peeters W Pauwels M Coucke W Lijnen A Effect of different mouthrinses on morning breath Journal of periodontology 2001 72 9 1183 1191 10.1902/jop.2000.72.9.1183 11577950

[B23] Shinada K Ueno M Konishi C Takehara S Yokoyama S Zaitsu T Effects of a mouthwash with chlorine dioxide on oral malodor and salivary bacteria a randomized placebo-controlled 7-day trial Trials 2010 12,11 1 14 10.1186/1745-6215-11-14 PMC2831889 20152022

[B24] Sopapornamorn P Ueno M Shinada K Vachirarojpisan T Kawaguchi Y Clinical application of a VSCs monitor for oral malodour assessment Oral health and preventive dentistry 2006 4 2 91 97 16813137

[B25] Rosenberg M Kulkarni GV Bosy A McCulloch CA Reproducibility and sensitivity of oral malodor measurements with a portable sulphide monitor Journal of dental research 1991 70 11 1436 1440 10.1177/00220345910700110801 1960254

[B26] Rosenberg M McCulloch CA Measurement of oral malodor: current methods and future prospects Journal of periodontology 1992 63 9 776 782 1474479 10.1902/jop.1992.63.9.776

[B27] Rosenberg M Septon I Eli I Bar-Ness R Gelernter I Brenner S Halitosis measurement by an industrial sulphide monitor Journal of periodontology 1991 62 8 487 489 1920015 10.1902/jop.1991.62.8.487

[B28] Tonzetich J Production and origin of oral malodor: a review of mechanisms and methods of analysis Journal of periodontology 1977 48 1 13 20 10.1902/jop.1977.48.1.13 264535

[B29] Oral health surveys. basic methods WHO 1997 4 Geneva

[B30] Silness J Loe H Periodontal Disease in Pregnancy. II. Correlation between Oral Hygiene and Periodontal Condition Acta odontologica Scandinavica 1964 22 121 135 10.3109/00016356408993968 14158464

[B31] Bez Y Coskun E Erol K Cingu AK Eren Z Topcuoglu V Adult strabismus and social phobia: a case-controlled study Journal of American Association for Pediatric Ophthalmology and Strabismus 2009 13 3 249 252 10.1016/j.jaapos.2009.02.010 19541264

[B32] Ballenger JC Davidson JR Lecrubier Y Nutt DJ Bobes J Beidel DC Consensus statement on social anxiety disorder from the International Consensus Group on Depression and Anxiety The Journal of clinical psychiatry 1998 59 Suppl 17 54 60 9811431

[B33] Bobes J Badia X Luque A Garcia M Gonzalez MP Dal-Re R Validation of the Spanish version of the Liebowitz social anxiety scale, social anxiety and distress scale and Sheehan disability inventory for the evaluation of social phobia Medicina clínica 1999 24,112 14 530 538 10363239

[B34] Yao SN Note I Fanget F Albuisson E Bouvard M Jalenques I Social anxiety in patients with social phobia: validation of the Liebowitz social anxiety scale: the French version L'Encéphale 1999 25 5 429 435 10598306

[B35] Turk CL Heimberg RG Orsillo SM Holt CS Gitow A Street LL An investigation of gender differences in social phobia Journal of anxiety disorders 1998 12 3 209 223 10.1016/S0887-6185(98)00010-3 9653680

[B36] Mannuzza S Schneier FR Chapman TF Liebowitz MR Klein DF Fyer AJ Generalized social phobia. Reliability and validity Archives of general psychiatry 1995 52 3 230 237 7872851 10.1001/archpsyc.1995.03950150062011

[B37] Weinshenker NJ Goldenberg I Rogers MP Goisman RM Warshaw MG Fierman EJ Profile of a large sample of patients with social phobia: comparison between generalized and specific social phobia Depression and anxiety 1996 4 5 209 216 10.1002/(SICI)1520-6394(1996)4:5<209::AID-DA1>3.0.CO;2-8 9167786

[B38] Rosenberg M Kozlovsky A Wind Y Mindel E Self-assessment of oral malodor 1 year following initial consultation Quintessence international 1999 30 5 324 327 10635287

[B39] Heimberg RG Cognitive-behavioral therapy for social anxiety disorder: current status and future directions Biological psychiatry 2002 1,51 1 101 108 10.1016/s0006-3223(01)01183-0 11801235

[B40] Liebowitz MR Heimberg RG Schneier FR Hope DA Davies S Holt CS Cognitive-behavioral group therapy versus phenelzine in social phobia: long-term outcome Depression and anxiety 1999 10 3 89 98 10.1002/(SICI)1520-6394(1999)10:3<89::AID-DA1>3.0.CO;2-5 10604081

[B41] Fedoroff IC Taylor S Psychological and pharmacological treatments of social phobia: a meta-analysis Journal of clinical psychopharmacology 2001 21 3 311 324 10.1097/00004714-200106000-00011 11386495

[B42] Kelsey JE Venlafaxine in social phobia Psychopharmacology bulletin 1995 31 4 767 771 8851651

[B43] Liebowitz MR Stein MB Tancer M Carpenter D Oakes R Pitts CD A randomized, double-blind, fixed-dose comparison of paroxetine and placebo in the treatment of generalized social anxiety disorder The Journal of clinical psychiatry 2002 63 1 66 74 10.4088/JCP.v63n0113 11838629

[B44] Stein MB Chavira DA Subtypes of social phobia and comorbidity with depression and other anxiety disorders Journal of affective disorders 1998 50 Suppl 1 11 16 9851573 10.1016/s0165-0327(98)00092-5

[B45] Sakurai A Nagata T Harai H Yamada H Mohri I Nakano Y Is "relationship fear" unique to Japan? Symptom factors and patient clusters of social anxiety disorder among the Japanese clinical population Journal of Affective Disorders 2005 87 1 131 137 10.1016/j.jad.2005.03.003 15894382

